# Long-term, patient-centered, frailty-based outcomes of older critical illness survivors from the emergency department: a post hoc analysis of the LIFE Study

**DOI:** 10.1186/s12877-024-04881-x

**Published:** 2024-03-15

**Authors:** Takashi Hongo, Tetsuya Yumoto, Mototaka Inaba, Shunsuke Taito, Takashi Yorifuji, Atsunori Nakao, Hiromichi Naito, the LIFE Study Investigators, Chikaaki Nakamichi, Hiroki Maeyama, Hideki Ishikawa, Nobuaki Shime, Sadayori Uemori, Satoshi Ishihara, Makoto Takaoka, Tsuyoshi Ohtsuka, Masahiro Harada, Satoshi Nozaki, Keisuke Kohama, Ryota Sakurai, Shuho Sato, Shun Muramatsu, Kazunori Yamashita, Toshihiko Mayumi, Kaoruko Aita, Satoshi Mochizuki, Hirofumi Itoh, Asase Senda, Kana Otani, Chison Gon, Takeshi Ohnishi, Yuji Taguchi, Toru Miike, Koki Umeda, Yuji Kondo, Takao Arai, Junya Tsurukiri

**Affiliations:** 1 Department of Emergency, Critical Care, and Disaster Medicine, Faculty of Medicine, Okayama University Graduate School of Medicine, Dentistry, and Pharmaceutical Sciences, Okayama University, 2-5-1 Shikata-cho, Kita-ku, Okayama, 700-8558 Japan; 2 Department of Clinical Practice and Support, Hiroshima University Hospital, 1-2-3 Minamiku Kasumi, Hiroshima, 734-0037 Japan; 3 Department of Epidemiology, Okayama University Graduate School of Medicine, Dentistry, and Pharmaceutical Sciences, 2-5-1 Shikata-cho, Kita-ku, Okayama, 700-8558 Japan

**Keywords:** ADL, Clinical frailty scale, Critical illness, Emergency department, Intensive care, QOL

## Abstract

**Background:**

Evidence indicates frailty before intensive care unit (ICU) admission leads to poor outcomes. However, it is unclear whether quality of life (QOL) and activities of daily living (ADL) for survivors of critical illness admitted to the ICU via the emergency department remain consistent or deteriorate in the long-term compared to baseline. This study aimed to evaluate long-term QOL/ADL outcomes in these patients, categorized by the presence or absence of frailty according to Clinical Frailty Scale (CFS) score, as well as explore factors that influence these outcomes.

**Methods:**

This was a post-hoc analysis of a prospective, multicenter, observational study conducted across Japan. It included survivors aged 65 years or older who were admitted to the ICU through the emergency department. Based on CFS scores, participants were categorized into either the not frail group or the frail group, using a threshold CFS score of < 4. Our primary outcome was patient-centered outcomes (QOL/ADL) measured by the five-level EuroQol five-dimensional questionnaire (EQ-5D-5L) and the Barthel Index six months post-ICU admission, comparing results from baseline. Secondary outcomes included exploration of factors associated with QOL/ADL six months post-ICU admission using multiple linear regression analyses.

**Results:**

Of 514 candidates, 390 participants responded to the EQ-5D-5L questionnaire, while 237 responded to the Barthel Index. At six months post-admission, mean EQ-5D-5L values declined in both the not frail and frail groups (0.80 to 0.73, *p* = 0.003 and 0.58 to 0.50, *p* = 0.002, respectively); Barthel Index scores also declined in both groups (98 to 83, *p* < 0.001 and 79 to 61, *p* < 0.001, respectively). Multiple linear regression analysis revealed that baseline frailty (β coefficient, -0.15; 95% CI, − 0.23 to − 0.07; *p* < 0.001) and pre-admission EQ-5D-5L scores (β coefficient, 0.14; 95% CI, 0.02 to 0.26; *p* = 0.016) affected EQ-5D-5L scores at six months. Similarly, baseline frailty (β coefficient, -12.3; 95% CI, − 23.9 to − 0.80; *p* = 0.036) and Barthel Index scores (β coefficient, 0.54; 95% CI, 0.30 to 0.79; *p* < 0.001) influenced the Barthel Index score at six months.

**Conclusions:**

Regardless of frailty, older ICU survivors from the emergency department were more likely to experience reduced QOL and ADL six months after ICU admission compared to baseline.

**Supplementary Information:**

The online version contains supplementary material available at 10.1186/s12877-024-04881-x.

## Background

The number of older patients visiting the emergency department is rising, with a subset of these individuals requiring critical care in the intensive care unit (ICU). This trend mirrors the dramatic increase in the older population [1–3]. Frailty in older individuals is characterized by weakness, reduced muscle mass, decreased mobility, diminished cognitive function, and poor nutritional status. The Clinical Frailty Scale (CFS) offers a flexible approach to evaluating frailty, considering a wide array of health deficits to assess frailty as a spectrum [4]. Elevated CFS scores have been linked with increased mortality and adverse outcomes, suggesting it as a suitable tool for ICU triage [5]. The LIFE Study further highlighted that CFS score is an independent predictor of long-term mortality among critically ill older patients admitted to the ICU through the emergency department in Japan [6]. The implications of this study are beneficial to clinicians, offering patients and their surrogates vital prognostic information about survival. Yet, long-term patient-centered outcomes such as quality of life (QOL), activities of daily living (ADL), return to home, and mental health outcomes are also significant concerns despite limited evidence [7, 8]. Tools such as the five-level EuroQol five-dimensional questionnaire (EQ-5D-5L) [9] and the Barthel Index [10], which measure QOL and ADL, respectively, should be considered in healthcare resource allocation decisions. While clinicians often predict future QOL/ADL based on their clinical experience without objective indicators, a poor CFS score before ICU admission might predict unfavorable outcomes [11, 12]. It remains unclear whether QOL/ADL in critical illness survivors remains unchanged or deteriorates in the long-term compared to baseline, as well as which factors are associated with long-term, patient-centered outcomes.

Therefore, this study aimed to investigate the long-term, patient-centered outcomes of ICU survivors expressed as a composite EQ-5D-5L/Barthel Index score grouped by frailty based on CFS score compared to baseline. Additionally, we evaluated the impact of this composite score on the QOL/ADL of these patients in the long term and explored the factors associated with QOL/ADL.

## Methods

### Study protocol

We conducted a post hoc analysis of the LIFE Study, which was registered in the University Hospital Medical Information Network Clinical Trials Registry (ID: UMIN000037430, date of registration: July 20, 2019), focusing on the QOL/ADL of LIFE Study survivors at six months post-ICU admission. The detailed study design and methods have been previously reported [6]. In brief, the LIFE Study was a prospective, multicenter, observational study conducted in 17 Japanese ICUs. Its primary objective was to determine whether baseline frailty impacted six-month mortality following ICU admission. All participants aged 65 years or older who were admitted to the ICU through the emergency department, including those requiring emergency surgery, between November 2019 and April 2020 were eligible for the LIFE Study analysis. The exclusion criteria included patients who did not provide consent, those without CFS data before ICU admission, and those lost to follow-up. In this post hoc analysis, we further excluded patients who died within six months and those lacking patient-centered data such as EQ-5D-5L and Barthel Index scores at both ICU admission and at the six-month follow-up.

The original study protocol was approved by the ethics committees of all participating institutions. Additionally, our post hoc analysis received approval from the Okayama University Hospital Ethics Committee (approval number: K-2308-008). Clinical investigations were conducted in accordance with the provisions of the Declaration of Helsinki. Patient consent was waived for all participants enrolled in this study by the Okayama University Hospital Ethics Committee, as the consent had already been obtained, and the data were analyzed anonymously.

### Assessment of frailty, QOL and ADL

On ICU admission, CFS scores were used to assess the frailty of all participating patients as they presented prior to the onset of the acute illness/injury (approximately two weeks earlier). The CFS is a pictographic scale that ranges from 1 (very fit) to 9 (terminally ill) [4]. Patients with scores of 1 to 3 are considered “not frail,” those with a score of 4 are termed “pre-frail” or “vulnerable,” and those with scores of 5 to 9 are considered “frail” [13]. Additionally, eligible participants completed questionnaires prior to ICU admission to assess baseline QOL and ADL. QOL was measured using the EQ-5D-5L, a general instrument that measures five dimensions: self-care, mobility, activities, anxiety/depression, and pain/discomfort. Each dimension is measured with scores ranging from 1 (no problems) to 5 (extreme problems) [9]. Specific EQ-5D-5L scores calculated using country-specific scores, with 1 indicating the best possible QOL and 0 indicating death [9]. The Barthel Index was utilized to evaluate the level of dependency in ADL. The Barthel Index encompasses ten domains: feeding, movement, adjustment, bathing, toilet use, mobility, dressing, stairs, bladder control, and bowel control, with possible scores of 0, 5, 10, or 15 points for each domain [10]. A higher Barthel Index score, ranging from 0 to 100 points, indicates a lower level of dependency. In a similar manner, a follow-up survey was conducted six months post-ICU admission to evaluate these patient-centered outcomes, again assessed using the EQ-5D-5L and Barthel Index. Upon ICU admission, trained study investigators obtained CFS and Barthel Index scores from either the patient or their surrogate. In addition, a survey to gather EQ-5D-5L score was administered to the patient or surrogate. Six months post-admission, a second questionnaire was mailed to patients and/or their surrogates to collect data on both the EQ-5D-5L and Barthel Index. Of note, Barthel Index data was collected from patients who were included since January 2020.

### Data collection

We collected the following data: patient characteristics (gender, age, body mass index, Charlson Comorbidity Index score, and ICU admission category such as cardiology, pulmonary, gastrointestinal, neurology, trauma, endocrine, skin/tissue, urology, or other); ICU admission type (medical or surgical); and illness severity (based on Acute Physiology and Chronic Health Evaluation (APACHE) 2 score on the first day after ICU admission), laboratory findings (specifically, maximum lactate concentrations during ICU admission); procedures during ICU admission (including mechanical ventilation, tracheostomy, renal replacement therapy, vasopressor support, and extracorporeal membrane oxygenation); presence of conditions such as sepsis or acute kidney injury during ICU admission; and outcome measures (such as discharge destination and length of ICU and hospital stay).

### Outcomes

Participants were categorized into two groups for analysis. The “not frail” and “frail” groups were defined based on the presence or absence of frailty, determined by a CFS score of 4 or greater before ICU admission [14]. Our primary outcome was patient-centered outcomes (specifically QOL/ADL assessed by EQ-5D-5L and Barthel Index) six months after ICU admission, comparing results from baseline, assigned by the not frail and frail groups.

Secondary outcomes included distinct functional capacities in QOL/ADL for both the not frail group and the frail group. Furthermore, we evaluated the impact of frailty on QOL/ADL six months after ICU admission and identified the factors associated with these changes.

### Statistical analyses

Categorical variables are presented as counts and percentages, while continuous variables are summarized using medians and interquartile ranges (IQRs) or means and standard deviations (SD). Changes in QOL/ADL scores from baseline to six months post-ICU admission are presented as mean differences and were analyzed using the paired-sample Student’s t-test. To compare between groups, the Mann-Whitney U test was used for continuous variables and Fisher’s exact test for categorical variables. A negative mean difference in EQ-5D-5L values and Barthel Index scores indicates worsening of QOL/ADL.

To assess the impact of CFS score on patient outcomes, multiple linear regression analyses were employed to estimate adjusted effects on QOL/ADL. These models controlled for variables such as gender, age, Charlson Comorbidity Index, body mass index, ICU admission category, ICU admission type, APACHE2 score, maximum lactate levels, presence of acute kidney injury, sepsis, length of ICU stay, and whether the patient was in the not frail or the frail group, as well as their EQ-5D-5L or Barthel Index scores prior to admission. In the model, variables with missing data, including APACHE2 score and lactate levels, were treated as missing. Results were presented as β coefficients with 95% confidence intervals (CIs).

We also conducted two sensitivity analyses. Firstly, we used an alternative definition for “frailty” based on a previous study [7], defining it as a CFS score of 5 or greater [14]. Secondly, we analyzed CFS as a continuous variable.

In addition, we compared the characteristics of patients who had complete EQ-5D-5L or Barthel Index data at both admission and 6 months with those who did not, to examine potential differences between them. For the Barthel Index analysis, we included patients admitted on or after January 1, 2020, which is when we began collecting this data.

A *p*-value of < 0.05 was considered statistically significant. All statistical analysis was performed using Stata version 17 (StataCorp LP, College Station, TX).

## Results

**Fig. 1:**
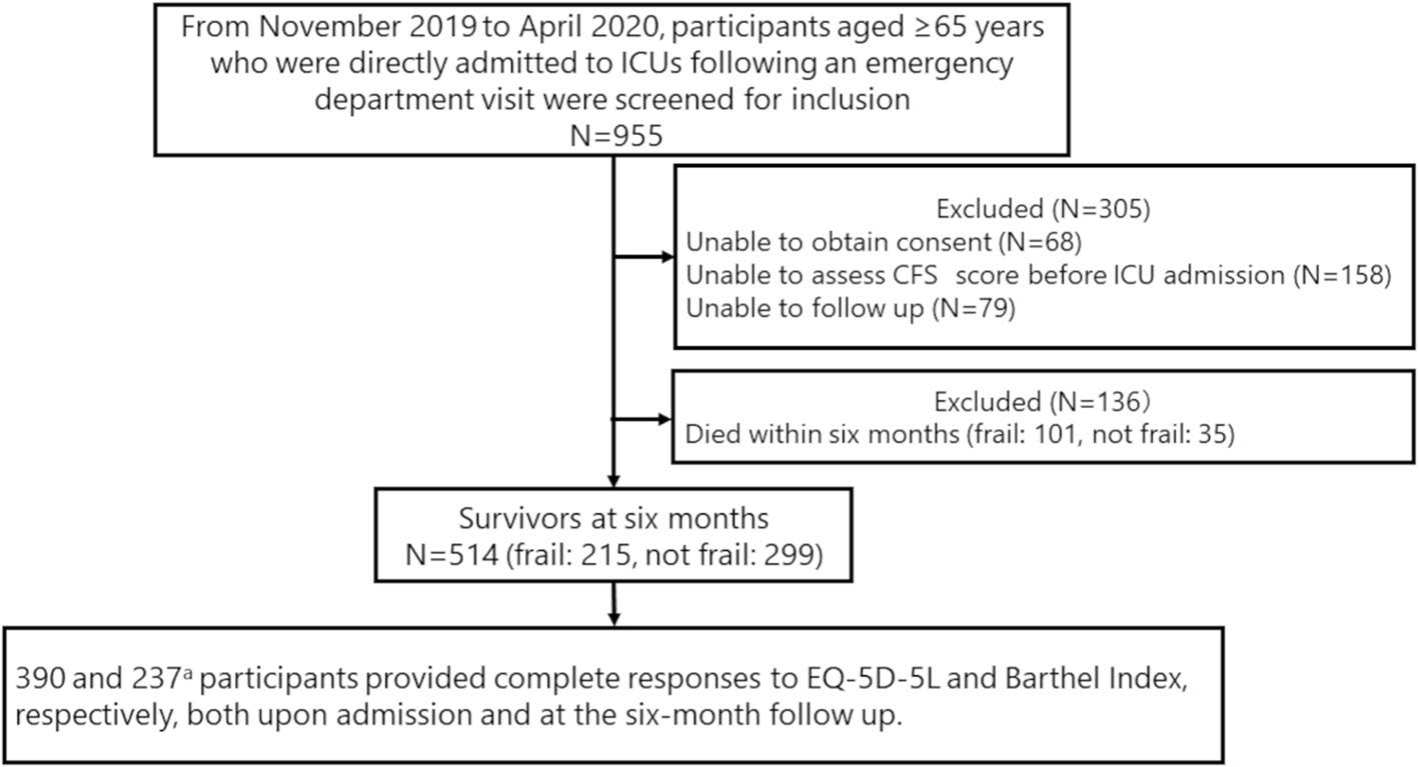
Flow chart of our study. ICU: intensive care unit, CFS: Clinical Frailty Scale, EQ-5D-5L: five-level EuroQol five-dimensional questionnaire. ^a^ Participants were included during the four-month period from January 2020 to April 2020

During the six-month period, 955 older participants admitted to ICUs via an emergency department visit were identified. After excluding specific cases (*n* = 305) and those who died within six months (*n* = 136), 514 participants (68.5%) had survived the six-month period. Of these, 390 participants provided complete responses to the EQ-5D-5L. Meanwhile, during a four-month time period, 237 participants provided Barthel Index responses (Fig. 1).

Ultimately, of the EQ-5D-5L responders, 242 participants were assigned to the not frail group and 148 to the frail group. For the Barthel Index responders, 132 were categorized into the not frail group and 105 into the frail group. The demographics and clinical characteristics of the participants are presented in Table 1. No patients with CFS scores of 8 or 9 were categorized.

**Table 1:** Patient characteristics of the study population

	Survivors at six months post-ICU admission (*n* = 514)			EQ-5D-5L responders both on admission and at six months (*n* = 390)			Barthel Index responders both on admission and at six months (*n* = 237)		
	All	Not Frail ^a^ (*n* = 299)	Frail ^b^ (*n* = 215)	All	Not Frail (*n* = 242)	Frail (*n* = 148)	All	Not Frail (*n* = 132)	Frail (*n* = 105)
Age, median (IQR), y	78 (71–84)	75 (70–81)	81 (75–87)	77 (71–83)	75 (71–81)	80 (75–86)	78 (71–83)	75 (70–81)	80 (74–85)
Body mass index, median (IQR) ^c^	22.0 (19.5–24.1)	22.2 (20.0-24.2)	21.2 (18.7–23.9)	21.9 (19.4–24.0)	22.1 (20.2–24.2)	21.0 (18.3–23.5)	21.9 (19.4–24.0)	22.1 (20.1–24.0)	21.3 (18.5–24.1)
Gender, n (%)									
Male	293 (57.0)	189 (63.2)	104 (48.4)	231 (59.2)	161 (66.5)	70 (47.2)	138 (58.2)	87 (65.9)	51 (48.6)
Female	221 (43.0)	110 (36.8)	111 (51.6)	159 (40.8)	81 (33.4)	78 (52.7)	99 (41.8)	45 (34.1)	54 (51.4)
Charlson Comorbidity Index score, median (IQR)	4 (4–6)	4 (3–5)	5 (4–6)	4 (3–6)	4 (3–5)	5 (4–6)	5 (4–6)	4 (3–5)	5 (4–6)
Cerebrovascular disease, n (%)	67 (13.0)	30 (10.0)	35 (16.2)	45 (11.5)	23 (9.5)	22 (14.8)	32 (13.5)	14 (10.6)	18 (17.1)
Chronic heart failure, n (%)	49 (9.5)	20 (6.6)	29 (13.4)	35 (8.9)	18 (7.4)	17 (11.4)	18 (7.5)	6 (4.5)	12 (11.4)
Chronic kidney disease, n (%)	27 (5.2)	8 (2.6)	19 (8.8)	19 (4.8)	7 (2.8)	12 (8.1)	15 (6.3)	6 (4.5)	9 (8.5)
Diabetes, n (%)	93 (18.0)	50 (16.7)	43 (20.0)	69 (17.6)	43 (17.7)	26 (17.5)	48 (20.2)	27 (20.4)	21 (20.0)
Malignancy, n (%)	76 (14.7)	34 (11.3)	36 (16.7)	52 (13.3)	28 (7.4)	24 (16.2)	39 (16.4)	20 (15.1)	19 (18.0)
Dementia, n (%)	47 (9.1)	4 (2.3)	43 (20.0)	32 (8.2)	1 (0.4)	31 (20.9)	19 (8.0)	2 (1.5)	17 (16.1)
CFS score, median (IQR)	3 (2–4)	3 (1–3)	5 (4–6)	3 (2–4)	3 (1–3)	4 (4–6)	3 (2–4)	3 (1–3)	4 (4–6)
1, n (%)	76 (14.7)	76 (25.4)	0 (0)	64 (16.4)	64 (26.4)	0 (0)	39 (16.4)	39 (29.5)	0 (0)
2, n (%)	57 (11.0)	57 (19.0)	0 (0)	47 (12.0)	47 (19.4)	0 (0)	25 (10.5)	25 (18.9)	0 (0)
3, n (%)	166 (32.2)	166 (55.5)	0 (0)	131 (33.5)	131 (54.1)	0 (0)	68 (29.1)	68 (51.5)	0 (0)
4, n (%)	104 (20.2)	0 (0)	104 (48.3)	79 (20.2)	0 (0)	79 (53.3)	54 (22.7)	0 (0)	54 (51.4)
5, n (%)	40 (7.7)	0 (0)	40 (18.6)	26 (6.6)	0 (0)	26 (17.5)	17 (7.1)	0 (0)	17 (16.1)
6, n (%)	39 (9.0)	0 (0)	39 (18.1)	21 (5.3)	0 (0)	21 (14.1)	16 (6.7)	0 (0)	16 (15.2)
7, n (%)	32 (6.2)	0 (0)	32 (14.8)	22 (5.6)	0 (0)	22 (14.8)	18 (7.5)	0 (0)	18 (17.1)
8 or 9, n (%)	0 (0)	0 (0)	0 (0)	0 (0)	0 (0)	0 (0)	0 (0)	0 (0)	0 (0)
ICU admission category, n (%)									
Cardiology	121 (23.5)	70 (23.4)	51 (23.7)	94 (24.8)	58 (23.9)	36 (24.3)	51 (21.5)	26 (19.6)	25 (23.8)
Pulmonary	47 (9.1)	17 (5.6)	30 (13.9)	34 (8.7)	14 (5.7)	20 (13.5)	26 (10.9)	11 (8.3)	15 (14.2)
Gastrointestinal	76 (14.7)	43 (14.3)	33 (15.3)	53 (13.5)	28 (11.5)	25 (16.8)	41 (17.2)	15 (11.3)	16 (15.2)
Neurology	124 (24.1)	79 (26.4)	45 (20.9)	93 (23.8)	66 (27.2)	27 (18.2)	61 (25.7)	39 (29.5)	22 (20.9)
Trauma	78 (15.1)	61 (20.4)	17 (7.9)	64 (16.4)	52 (21.4)	12 (8.1)	40 (16.8)	29 (21.9)	11 (10.4)
Endocrine	23 (4.4)	10 (3.3)	13 (6.0)	19 (4.8)	8 (3.3)	11 (7.4)	11 (4.6)	5 (3.7)	6 (5.7)
Skin/tissue	6 (1.1)	3 (1.0)	3 (1,3)	5 (1.2)	3 (1.2)	2 (1.3)	2 (1.4)	1 (0.7)	1 (0.9)
Urology	5 (0.9)	2 (0.6)	3 (1.3)	3 (0.7)	2 (0.8)	1 (0.6)	2 (1.4)	1 (0.7)	1 (0.9)
Others	34 (6.6)	14 (4.6)	20 (9.3)	25 (6.4)	11 (4.5)	14 (9.4)	13 (5.4)	5 (3.7)	8 (7.6)
Admission type, n (%)									
Medical	313 (60.9)	171 (57.2)	142 (66.0)	237 (60.7)	137 (56.6)	100 (67.5)	143 (60.3)	74 (56.0)	69 (65.7)
Surgical	201 (39.1)	128 (42.8)	73 (33.9)	153 (39.2)	105 (43.3)	48 (32.4)	94 (39.7)	58 (44.0)	36 (32.3)
APACHE2 score, median (IQR) ^d^	20 (15–26)	18 (14–24)	22 (17–28)	19 (15–25)	17 (13–24)	22 (17–28)	20 (16–27)	18 (14–25)	23 (18–31)
Maximum lactate levels during ICU stay, median (IQR) mol/L ^e^	2.1 (1.3–3.9)	1.9 (1.2–3.7)	2.4 (1.6–4.9)	2.1 (1.3–3.9)	1.9 (1.2–3.5)	2.5 (1.6–5.2)	2.1 (1.4–4.1)	1.9 (1.3–3.3)	2.7 (1.7–5.1)
Sepsis, n (%)	59 (11.4)	24 (8.0)	35 (16.2)	43 (11.0)	19 (7.8)	24 (16.2)	28 (11.8)	11 (8.3)	17 (16.1)
Acute kidney injury, n (%)^f^	92 (17.9)	43 (14.3)	49 (22.7)	73 (18.7)	38 (15.7)	35 (23.6)	53 (22.3)	24 (18.1)	29 (27.6)
Mechanical ventilation, n (%)	183 (35.6)	96 (32.1)	87 (40.4)	134 (34.3)	78 (32.2)	56 (47.8)	96 (40.5)	48 (36.3)	48 (45.7)
Tracheostomy, n (%)	29 (5.6)	16 (5.3)	13 (6.0)	17 (4.3)	12 (4.9)	5 (3.3)	12 (5.0)	5 (3.7)	7 (6.6)
Vasopressor support, n (%)	433 (84.2)	260 (86.9)	173 (80.4)	332 (85.1)	210 (86.7)	122 (82.4)	195 (82.2)	110 (8.3)	85 (80.9)
Renal replacement therapy, n (%)	36 (7.0)	15 (5.0)	21 (9.7)	26 (6.6)	13 (5.3)	13 (8.7)	18 (7.5)	8 (6.0)	10 (9.5)
ECMO, n (%)	9 (1.7)	7 (2.3)	2 (0.9)	8 (2.0)	6 (2.4)	2 (1.3)	5 (2.1)	4 (3.0)	1 (0.9)
ICU length of stay, median (IQR), days	3 (1–6)	3 (1–7)	2 (1–6)	3 (1–6)	3 (1–7)	2 (1–5)	3 (1–6)	4 (1–7)	3 (1–6)
Length hospital of stay, median (IQR), days ^g^	17 (9–29)	16 (9–27)	18 (9–31)	16 (8–28)	16 (8–27)	18 (7–31)	16 (8–29)	15 (8–28)	16 (8–31)
Discharged to home from the hospital, n (%) ^h^	380 (74.5)	250 (84.1)	130 (61.0)	293 (75.7)	201 (83.7)	92 (62.5)	169 (71.9)	106 (80.9)	63 (60.5)
Responders on admission, n (%) ^i^									
Patients	132 (25.7)	97 (32.6)	35 (16.3)	108 (27.7)	81 (33.5)	27 (18.2)	69 (29.1)	48 (36.3)	21 (20.0)
Surrogates	381 (74.3)	201 (67.4)	180 (83.7)	282 (72.3)	161 (66.5)	121 (81.8)	168 (70.9)	84 (63.7)	84 (80.0)
Responders at six months, n (%) ^j^									
Patients	216 (42.2)	158 (53.0)	58 (27.1)	183 (46.9)	142 (58.7)	41 (27.7)	105 (44.3)	74 (56.1)	31 (29.5)
Surrogates	296 (57.8)	140 (47.0)	156 (72.9)	207 (53.1)	100 (41.3)	107 (72.3)	132 (55.7)	58 (33.9)	74 (70.5)

*ICU* Intensive care unit, *EQ-5D-5L* Five-level EuroQol five-dimensional questionnaire, *IQR* Interquartile range, *CFS* Clinical Frailty Scale, *APACHE2* Acute Physiology and Chronic Health Evaluation 2, *ECMO* Extracorporeal membrane oxygenation

^a^Defined as CFS score of 1, 2, or 3 before ICU admission

^b^Defined as CFS score of 4 or greater before ICU admission

^c^Of 514 participants, two were missing from the six-month survivors group, and one was missing from the EQ-5D-5L responders

^d^Of 514 participants, 130 were missing from the six-month survivors group, 92 from the EQ-5D-5L responders, and 98 from the Barthel Index responders

^e^Of 514 participants, 116 were missing from the six-month survivors group, 83 from the EQ-5D-5L responders, and 53 from the Barthel Index responders

^f^Of 514 participants, two were missing from the six-month survivors group, and one was missing from the EQ-5D-5L responders

^g^Of 514 participants, two were missing from the six-month survivors group, and one was missing from the EQ-5D-5L responders

^h^Of 514 participants, two were missing from the six-month survivors group, three from the EQ-5D-5L responders, and two from the Barthel Index responders

^i^Of 514 participants, one was missing from the six-month survivors

^j^Of 514 participants, two were missing from the six-month survivors

**Fig. 2:**
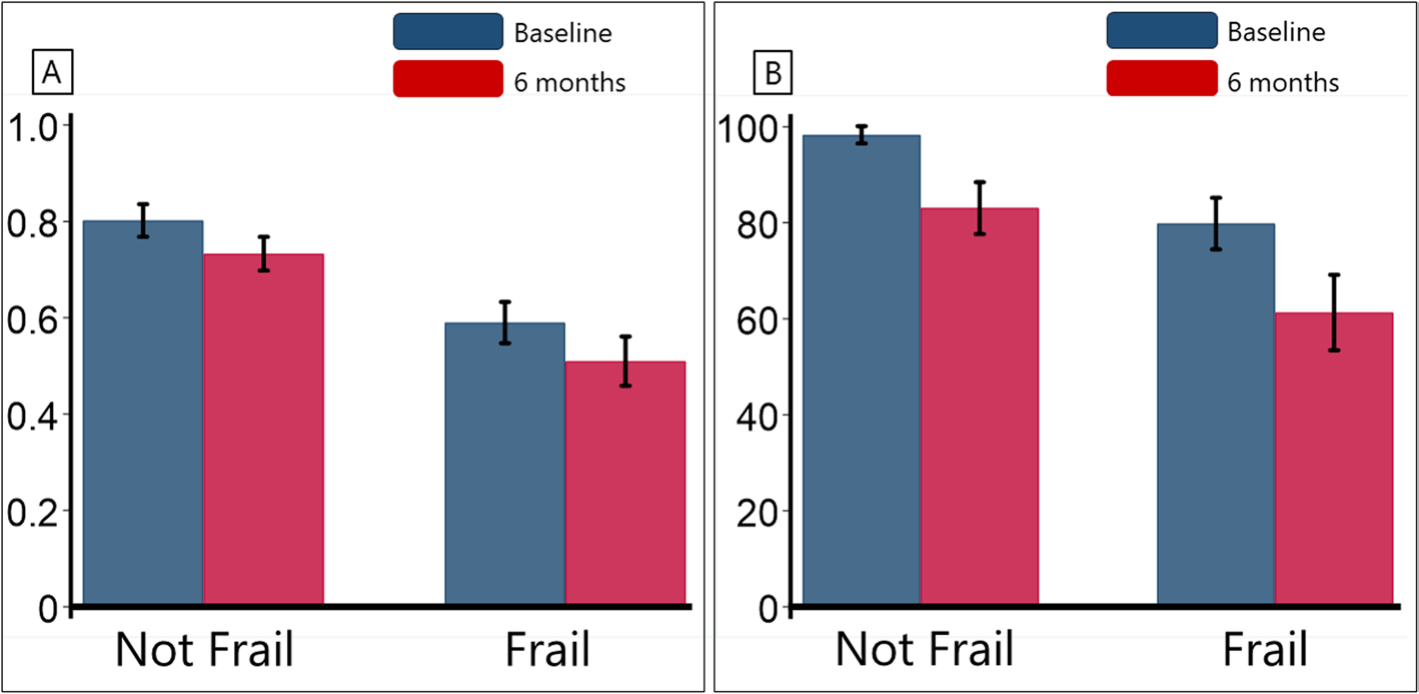
Comparison of mean EQ-5D-5L values (**A**) and Barthel Index scores (**B**) between baseline and six months, categorized by not frail and frail groups. EQ-5D-5L: five-level EuroQol five-dimensional questionnaire

The mean (SD) EQ-5D-5L scores decreased over six months for both not frail (0.80 [0.26] to 0.73 [0.27], *p* = 0.003) and frail groups (0.58 [0.26] to 0.50 [0.31], *p* = 0.002) (Fig. 2A). Similarly, Barthel Index scores dropped for not frail (98 [10] to 83 [31], *p* < 0.001) and frail (79 [27] to 61 [40], *p* < 0.001) (Fig. 2B). After stratification by each CFS score, both the EQ-5D-5L scores and Barthel Index scores showed a decreasing trend at six months compared to baseline (Additional file 1).

Differences in EQ-5D-5L scores between the not frail and frail groups are illustrated in a spider graph, capturing all five domains (Additional file 2). Regardless of the presence of frailty, the scores for mobility, self-care, and activities worsened significantly. However, unexpectedly, the scores for pain/discomfort in the frail group showed significant improvement. The differences in ADL between the not frail and the frail groups are summarized in a spider graph, depicting all 10 domains (Additional file 3). The mean scores for all domains, including feeding, movement, adjustment, bathing, toilet use, stairs, mobility, dressing, bladder control, and bowel control decreased from baseline to six months after admission for both the not frail and the frail groups.

Multiple linear regression analysis was conducted to determine the factors associated with the EQ-5D-5L and Barthel Index scores, respectively, at six months post-admission. The analysis demonstrated that frailty, based on CFS scores (1 to 3) at baseline before ICU admission (β coefficient, -0.15; 95% CI, − 0.23 to − 0.07; *p* < 0.001), APACHE2 score (β coefficient, -0.005; 95% CI, − 0.01 to − 0.002; *p* = 0.039), EQ-5D-5L score before ICU admission (β coefficient, 0.14; 95% CI, 0.02 to 0.26; *p* = 0.016), and length of ICU stay (β coefficient, -0.004; 95% CI, − 0.009 to − 0.0001; *p* < 0.001) were significantly associated with EQ-5D-5L scores at six months post-admission. Similarly, frailty (β coefficient, -12.3; 95% CI, − 23.9 to − 0.08; *p* = 0.036) and Barthel Index score before ICU admission (β coefficient, 0.54; 95% CI, 0.30 to 0.79; *p* < 0.001) were significantly associated with Barthel Index score at six months post-admission (Table 2).

**Table 2:** Factors associated with the EQ-5D-5L and Barthel Index scores at six months post-ICU admission

Variables	β coefficient (95% CI)	*P*-value
EQ-5D-5L score at six months		
Frailty	-0.15 (-0.23 to -0.07)	< 0.001
APACHE2 score	-0.005 (-0.01 to -0.002)	0.039
EQ-5D-5L score before admission	0.14 (0.02 to 0.26)	0.016
Length of ICU stay	-0.004 (− 0.009 to -0.001)	< 0.001
Barthel Index score at six months		
Frailty	-12.3 (-23.9 to -0.80)	0.036
Barthel Index score before admission	0.54 (0.30 to 0.79)	< 0.001

Variables included in the regression analysis for the outcomes were age, gender, body mass index, Charlson Comorbidity Index score, ICU admission category, ICU admission type, APACHE2 score, maximum lactate levels, presence of acute kidney injury, sepsis, length of ICU stay, frailty (based on Clinical Frailty Scale score ≥ 4), and either EQ-5D-5L or Barthel Index score. Only statistically significant variables are shown in the table

*EQ-5D-5L* Five-level EuroQol five-dimensional questionnaire, *CI* Confidence interval, *ICU* Intensive care unit, *APACHE2* Acute Physiology and Chronic Health Evaluation 2

In sensitivity analysis, frailty was associated with EQ-5D-5L and Barthel Index scores at six months post-ICU admission, even when using an alternative definition of frailty or treating it as a continuous variable (Additional files 4 and 5).

In additional analysis, the majority of patients with missing EQ-5D-5L or Barthel Index data were found to be missing this information at the 6-month follow-up, rather than at admission. Although the characteristics of patients with complete Barthel Index data and those with missing data were similar, patients with missing EQ-5D-5L data had higher CFS scores at admission (Additional file 6).

## Discussion

In this post hoc analysis of the LIFE Study, a prospective, multicenter cohort study conducted in Japan, we found that older patients admitted to the ICU via the emergency department typically experienced a decline in QOL/ADL at six months post-ICU admission, irrespective of their frailty status. The APACHE2 score, initial EQ-5D-5L score, and Barthel Index score before ICU admission were significant predictors of long term health related QOL and ADL. Moreover, longer ICU stays negatively correlated with QOL. This study provides pivotal insights that could influence decisions regarding ICU admissions for older patients, given the limited available evidence.

Frailty, as gauged by CFS, plays an instrumental role in predicting unfavorable outcomes post-ICU admission. Our results reinforce frailty’s robust link to diminished QOL (EQ-5D-5L scores) and ADL (Barthel Index scores) over a six-month post-ICU period [15–18]. A recent study assessed the risk of in-hospital death among critically ill patients with pneumonia using CFS scores upon admission [19]. The findings suggest that frailty alone might not be an effective criterion for predicting short-term mortality. Nonetheless, recognizing the significant differences between frail and non-frail individuals is essential, not just for immediate survival but also for enhancing longer-term, patient-centered outcomes [20]. Our study uniquely focused on older patients in emergency intensive care in Japan, a country with a notably high proportion of older individuals, emphasizing the importance of long-term care and QOL post-emergency. Both the not frail and frail groups demonstrated a marked decline in QOL and ADL scores six months after ICU admission. This suggests that while frailty exacerbates the decline, ICU admission due to acute illness or injury poses a substantial burden to older patients, even if they were not frail at onset.

In examining the distinct functional capacities in QOL/ADL for both the not frail and the frail groups, we observed a deterioration in physical function post-ICU admission. However, the anxiety/depression composite remained stable. These findings are consistent with those from previous studies indicating that these psychological symptoms can remain stable over time in ICU survivors [21, 22]. Unexpectedly, there was a noticeable improvement in pain/discomfort scores in the frail group. This improvement might be partly attributed to the possibility that scores on ICU admission were inflated by the surrogate, given the patient’s ICU admission due to an acute illness or injury. Additionally, it is proposed that frail individuals might have an increased pain tolerance or could be underreporting their pain [23].

Our study aligns with previous research, which found that factors such as the APACHE2 score and initial EQ-5D-5L and Barthel Index scores prior to ICU admission were significant predictors for declines in QOL and ADL, respectively [18, 24, 25]. This emphasizes the criticality of initial assessments in anticipating long-term outcomes. Furthermore, in line with prior studies [24, 25], a longer ICU stay was significantly negatively correlated with QOL scores, suggesting it can predict a decline in long-term QOL.

The strength of this study lies in its examination of the associations between frailty and long-term patient-centered outcomes originating from the emergency department. This provides invaluable data for making decisions about ICU admission and predicting long-term outcomes after discharge. However, several limitations should be acknowledged. First, the measurement of CFS and QOL/ADL in this study was based on self-reporting. Patients or their surrogates might not accurately estimate CFS, QOL, and ADL prior to critical illness due to recall bias. Second, while the response rate for EQ-5D-5L was 76%, the Barthel Index had a lower response rate of only 46%. This discrepancy was because we did not collect Barthel Index data during the first two months of the entire six-month study period. Nonetheless, the fact that patients with missing EQ-5D-5L data had higher CFS scores at admission suggests the possibility of potential selection bias, warranting consideration in the interpretation of our findings. Third, we did not record the details of clinical course between ICU discharge and six months after ICU admission. Additional unmeasured or potential confounders such as physical rehabilitation and the other further complications may exist. Indeed, post-hospitalization interventions could lead to a subjective improvement in QOL for patients at risk of post-intensive care syndrome [26]. Fourth, assessing outcomes at a single time point (i.e., six months) may overlook important trends. Fifth, source of infection, which would impact long-term outcomes, was not available. Finally, the study is specific to the Japanese demographic and might need validation in other cultural and ethnic settings.

Despite these limitations, our study offers invaluable insights into the impact of ICU admissions on the QOL and ADL of older patients. It underscores the critical role frailty plays in the deterioration of patient outcomes, regardless of whether patients were frail at the onset of their ICU stay. Moreover, our findings have potential implications for healthcare providers in patient or family counseling and tailoring post-ICU care for the older patients. Future research should also prioritize the prevention of long-term QOL/ADL deterioration.

## Conclusions

Our findings stress the importance of comprehensive assessments and shed light on the profound impacts on the long-term well-being of older patients, regardless of their baseline frailty. While ICU admissions undoubtedly save lives, post-ICU QOL/ADL for older patients, both frail and non-frail, should be a primary concern. Future research should prioritize the development of strategies that can mitigate the negative impacts of ICU admissions on the older patients, aiming for not just survival but also enhanced QOL/ADL post-ICU.

### Supplementary Information

**Supplementary Material 1.**

**Supplementary Material 2.**

**Supplementary Material 3.**

**Supplementary Material 4.**

**Supplementary Material 5.**

**Supplementary Material 6.**

## Data Availability

The datasets from this study are available from the corresponding author upon request.

## Abbreviations

ADL: Activities of daily living
APACHE: Acute Physiologic and Chronic Health Evaluation
CFS: Clinical Frailty Scale
CI: Confidence interval
EQ-5D-5L: Five-level EuroQol five-dimensional questionnaire
ICU: Intensive care unit
IQR: Interquartile range
QOL: Quality of life
SD: Standard deviations
