# Supplementary material for: Long-term, patient-centered, frailty-based outcomes of older critical illness survivors from the emergency department: a post hoc analysis of the LIFE Study
Source: BMC Geriatr. 2024 Mar 15;24:257. doi: 10.1186/s12877-024-04881-x (PMC10941380; doi:10.1186/s12877-024-04881-x)
Supplement: Supplementary file 1 — Supplementary Material 1. [file 12877_2024_4881_MOESM1_ESM.docx]

**Additional file 1.** Comparison of mean EQ-5D-5L values (A) and Barthel Index scores (B) between baseline and six months, stratified by CFS score.


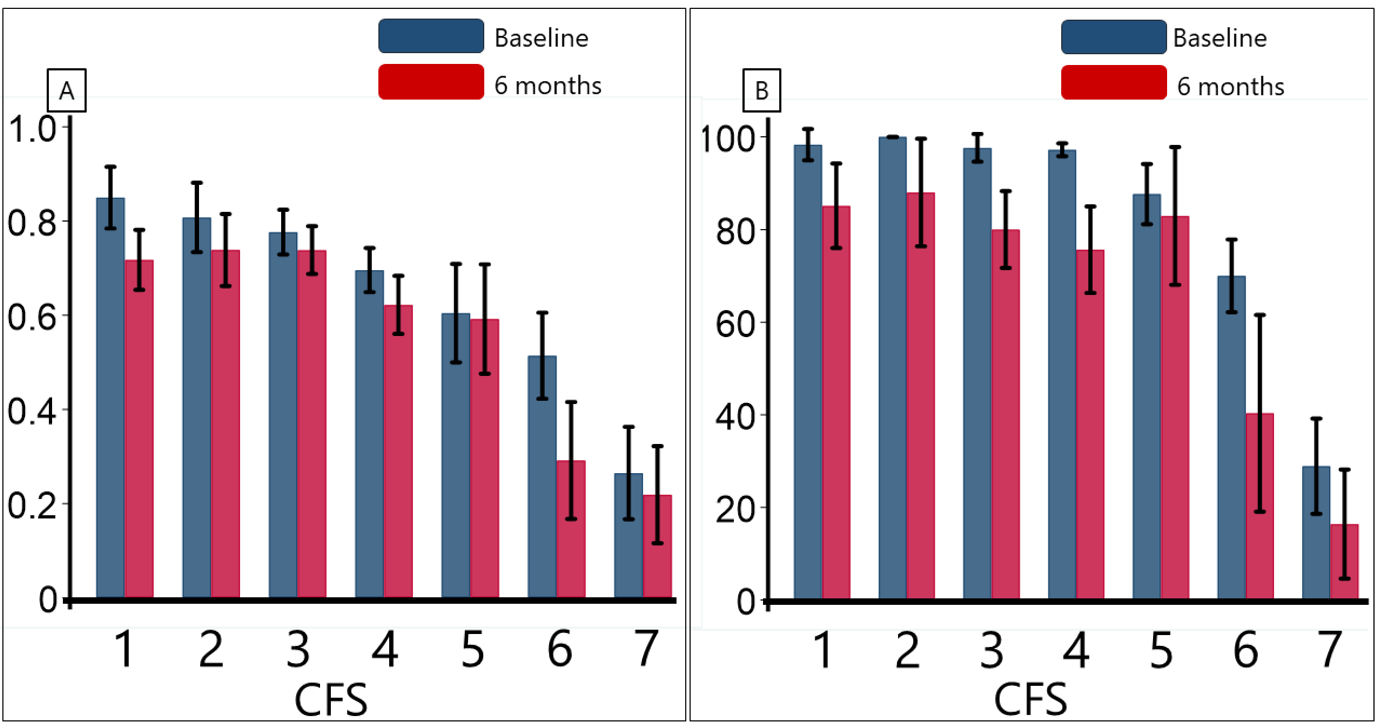


EQ-5D-5L: five-level EuroQol five-dimensional questionnaire, CFS: Clinical Frailty Scale
