# Supplementary material for: Long-term, patient-centered, frailty-based outcomes of older critical illness survivors from the emergency department: a post hoc analysis of the LIFE Study
Source: BMC Geriatr. 2024 Mar 15;24:257. doi: 10.1186/s12877-024-04881-x (PMC10941380; doi:10.1186/s12877-024-04881-x)
Supplement: Supplementary file 2 — Supplementary Material 2. [file 12877_2024_4881_MOESM2_ESM.docx]

**Additional file 2.** Differences in EQ-5D-5L for the not frail (A) and frail (B) groups: a spider graph depicting all five domains.


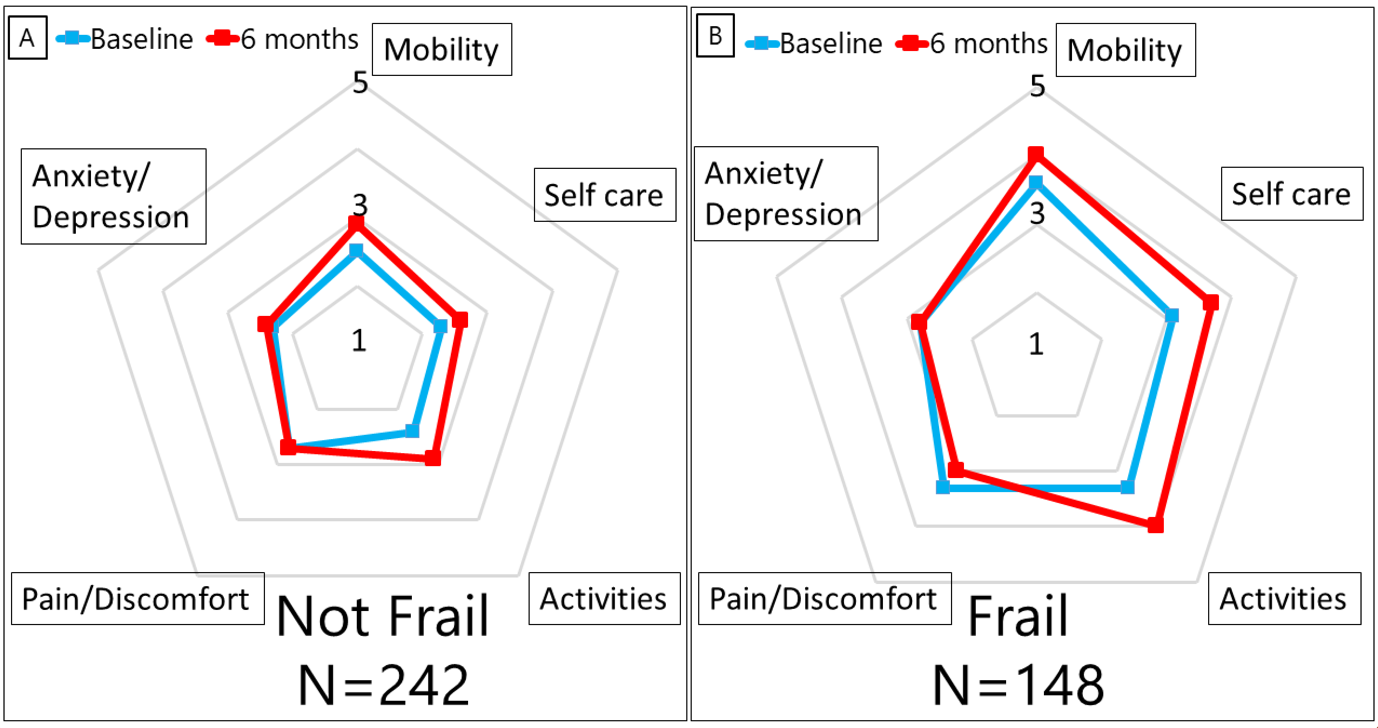


For the not frail group, the scores for mobility (1.5 vs. 1.9, p=0.001), self-care (1.3 vs. 1.6, p=0.009), and activities (1.4 vs. 1.9, p<0.001) worsened significantly, while the scores for pain/discomfort (1.7 vs. 1.7, p=0.71) and anxiety/depression (1.3 vs. 1.4, p=0.14) remained unchanged. For the frail group, there was significant worsening in the scores for mobility (2.6 vs. 3.0, p<0.001), self-care (2.1 vs. 2.7, p<0.001), and activities (2.3 vs. 3.0, p<0.001). However, the score for pain/discomfort significantly improved (2.3 vs. 2.0, p<0.001) and the score for anxiety/depression remained stable (1.8 vs. 1.8, p=1.00).

EQ-5D-5L: five-level EuroQol five-dimensional questionnaire
