# Supplementary material for: Long-term, patient-centered, frailty-based outcomes of older critical illness survivors from the emergency department: a post hoc analysis of the LIFE Study
Source: BMC Geriatr. 2024 Mar 15;24:257. doi: 10.1186/s12877-024-04881-x (PMC10941380; doi:10.1186/s12877-024-04881-x)
Supplement: Supplementary file 3 — Supplementary Material 3. [file 12877_2024_4881_MOESM3_ESM.docx]

**Additional file 3.** Differences in Barthel Index scores for the not frail (A) and frail (B) groups: a spider graph depicting all 10 domains.


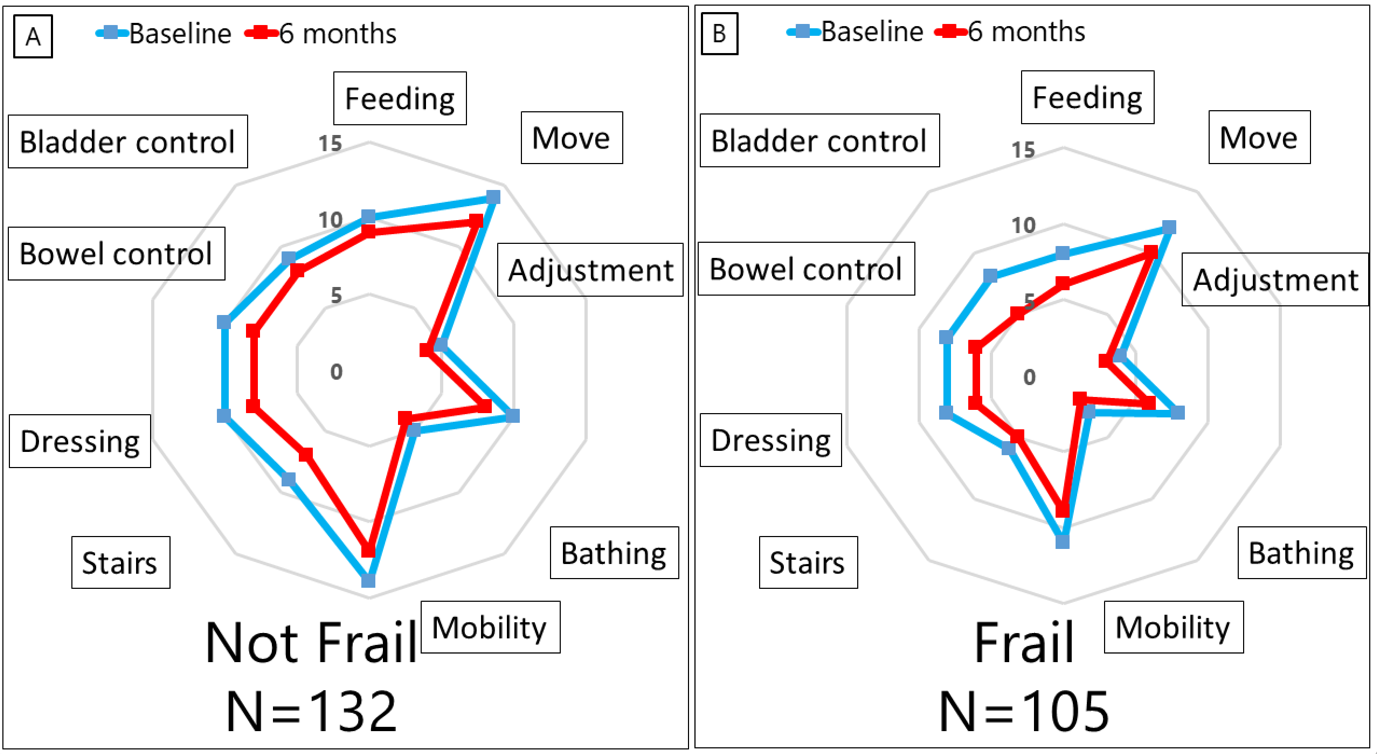


In both the not frail and frail groups, the scores for feeding (10 vs. 9, p<0.001) (8 vs. 6, p<0.001), movement (14 vs. 12, p<0.001) (12 vs. 10, p<0.001), adjustment (5 vs. 4, p<0.001) (4 vs. 3, p<0.001), toilet (10 vs. 8, p<0.001) (8 vs. 6, p<0.001), bathing (5 vs. 4, p<0.001) (3 vs. 2, p<0.001), mobility (14 vs. 12, p<0.001) (11 vs. 9, p<0.001), stairs (9 vs. 7, p=0.009) (6 vs. 5, p=0.002), dressing (10 vs. 8, p<0.001) (8 vs. 6, p<0.001), bowel control (10 vs. 8, p<0.001) (8 vs. 6, p<0.001), and bladder control (9 vs. 8, p<0.001) (8 vs. 5, p<0.001) worsened, respectively.
