# Supplementary material for: Long-term, patient-centered, frailty-based outcomes of older critical illness survivors from the emergency department: a post hoc analysis of the LIFE Study
Source: BMC Geriatr. 2024 Mar 15;24:257. doi: 10.1186/s12877-024-04881-x (PMC10941380; doi:10.1186/s12877-024-04881-x)
Supplement: Supplementary file 5 — Supplementary Material 5. [file 12877_2024_4881_MOESM5_ESM.docx]

**Additional file 5.** Factors associated with EQ-5D-5L and Barthel Index scores six months post-ICU admission, treating the Clinical Frailty Scale score as a continuous variable.

| **Variables** | **β coefficient (95% CI)** | **p-value** | **Variables** | **β coefficient (95% CI)** | **p-value** |
| --- | --- | --- | --- | --- | --- |
| EQ-5D-5L score at six months | | | Barthel Index score at six months | | |
| Clinical Frailty Scale score | -0.06 (-0.09 to -0.03) | <0.001 | Clinical Frailty Scale score | -7.08 (-11.1 to -2.99) | 0.001 |
| Length of ICU stay | -0.005 (-0.01 to -0.001) | <0.001 | Barthel Index score before admission | 0.34 (0.06 to 0.62) | 0.015 |

Variables included in the regression analysis for the outcomes were age, gender, body mass index, Charlson Comorbidity Index score, ICU admission category, ICU admission type, APACHE2 score, maximum lactate levels, presence of acute kidney injury, sepsis, length of ICU stay, Clinical Frailty Scale score, and either EQ-5D-5L or Barthel Index score. Additional file 5 showed statistically significant variables.

EQ-5D-5L, five-level EuroQol five-dimensional questionnaire; CI, confidence interval; ICU, intensive care unit; APACHE2, Acute Physiology and Chronic Health Evaluation 2
