# Supplementary material for: Long-term, patient-centered, frailty-based outcomes of older critical illness survivors from the emergency department: a post hoc analysis of the LIFE Study
Source: BMC Geriatr. 2024 Mar 15;24:257. doi: 10.1186/s12877-024-04881-x (PMC10941380; doi:10.1186/s12877-024-04881-x)
Supplement: Supplementary file 6 — Supplementary Material 6. [file 12877_2024_4881_MOESM6_ESM.docx]

**Additional file 6**. Comparison of patients with complete vs. missing EQ-5D-5L and Barthel Index data at admission and 6 months.

|  | **EQ-5D-5L completed at admission & 6 months**  **(n=390)** | **EQ-5D-5L missing at admission, 6 months, or both**  **(n=124)*** | **p-value** | **Barthel index completed at admission & 6 months**  **(n=237)** | **Barthel index missing at admission, 6 months, or both**  **(n=52)**** | **p-value** |
| --- | --- | --- | --- | --- | --- | --- |
| Frail (CFS ≥ 4), n (%) | 148 (37.9) | 67 (54.0) | 0.002 | 105 (44.3) | 21 (40.3) | 0.606 |
| Age, median (IQR) , y | 77 (71-83) | 78 (71-84) | 0.398 | 78 (71-83) | 76 (69-82) | 0.482 |
| Body mass index, median (IQR) ^a^ | 21.9 (19.4-24.0) | 22.1 (19.8-24.5) | 0.540 | 21.8 (19.8-24.4) | 22.0 (19.5-24.2) | 0.931 |
| Gender, n (%) |  |  | 0.071 |  |  | 0.278 |
| Men | 231 (59.2) | 62 (50.0) |  | 138 (58.2) | 26 (50.0) |  |
| Women | 159 (40.8) | 62 (50.0) |  | 99 (41.8) | 122 (50.0) |  |
| Charlson Comorbidity Index, median (IQR) | 4 (3-6) | 5 (4-6) | 0.078 | 5 (4-6) | 4 (3-5) | 0.528 |
| Cerebrovascular disease, n (%) | 45 (11.5) | 20 (16.1) | 0.180 | 32 (13.5) | 10 (19.2) | 0.288 |
| Chronic heart failure, n (%) | 35 (8.9) | 14 (11.2) | 0.444 | 18 (7.5) | 3 (5.7) | 0.646 |
| Chronic kidney disease, n (%) | 19 (4.8) | 8 (6.4) | 0.492 | 15 (6.3) | 3 (5.7) | 0.880 |
| Diabetes, n (%) | 69 (17.6) | 24 (19.3) | 0.675 | 48 (20.2) | 11 (21.1) | 0.884 |
| Malignancy, n (%) | 52 (13.3) | 18 (14.5) | 0.738 | 39 (16.4) | 7 (13.4) | 0.593 |
| Dementia, n (%) | 32 (8.2) | 15 (12.0) | 0.190 | 19 (8.0) | 6 (11.5) | 0.413 |
| CFS score, median (IQR) | 3 (2-4) | 4 (3-5) | <0.001 | 3 (2-4) | 3 (3-5) | 0.507 |
| 1, n (%) | 64 (16.4) | 12 (9.6) |  | 39 (16.4) | 6 (11.5) |  |
| 2, n (%) | 47 (12.0) | 10 (8.0) |  | 25 (10.5) | 3 (5.7) |  |
| 3, n (%) | 131 (33.5) | 35 (28.2) |  | 68 (29.1) | 22 (42.3) |  |
| 4, n (%) | 79 (20.2) | 25 (20.1) |  | 54 (22.7) | 6 (11.5) |  |
| 5, n (%) | 26 (6.6) | 14 (11.2) |  | 17 (7.1) | 6 (11.5) |  |
| 6, n (%) | 21 (5.3) | 18 (14.5) |  | 16 (6.7) | 5 (9.6) |  |
| 7, n (%) | 22 (5.6) | 10 (8.0) |  | 18 (7.5) | 4 (7.6) |  |
| 8 or 9, n (%) | 0 (0) | 0 (0) |  | 0 (0) | 0 (0) |  |
| ICU admission category, n (%) |  |  | 0.723 |  |  | 0.458 |
| Cardiology | 94 (24.8) | 27 (21.7) |  | 51 (21.5) | 8 (15.3) |  |
| Pulmonary | 34 (8.7) | 13 (10.4) |  | 26 (10.9) | 2 (3.8) |  |
| Gastrointestinal | 53 (13.5) | 23 (18.5) |  | 41 (17.2) | 10 (19.2) |  |
| Neurology | 93 (23.8) | 31 (25.0) |  | 61 (25.7) | 19 (36.5) |  |
| Trauma | 64 (16.4) | 14 (11.2) |  | 40 (16.8) | 6 (11.5) |  |
| Endocrine | 19 (4.8) | 4 (3.2) |  | 11 (4.6) | 3 (5.7) |  |
| Skin/tissue | 5 (1.2) | 1 (0.8) |  | 2 (1.4) | 0 (0) |  |
| Urology | 3 (0.7) | 2 (1.6) |  | 2 (1.4) | 1 (1.9) |  |
| Others | 25 (6.4) | 9 (7.2) |  | 13 (5.4) | 3 (5.7) |  |
| Admission type, n (%) |  |  | 0.918 |  |  | 0.171 |
| Medical | 237 (60.7) | 76 (61.3) |  | 143 (60.3) | 26 (50.0) |  |
| Surgical | 153 (39.2) | 48 (38.7) |  | 94 (39.7) | 26 (50.0) |  |
| APACHE2, median (IQR) ^b^ | 19 (15-25) | 21 (17-28) | 0.017 | 20 (16-27) | 20 (14-24) | 0.535 |
| Maximum lactate levels during the ICU stay, median (IQR) mol/L ^c^ | 2.1 (1.3-3.9) | 2.1 (1.3-4.0) | 0.901 | 2.1 (1.4-4.1) | 1.6 (1.2-3.6) | 0.214 |
| Sepsis, n (%) | 43 (11.0) | 16 (12.9) | 0.568 | 28 (11.8) | 4 (7.6) | 0.391 |
| Acute kidney injury, n (%) ^d^ | 73 (18.7) | 19 (15.3) | 0.403 | 53 (22.3) | 4 (7.6) | 0.016 |
| Mechanical Ventilation, n (%) | 134 (34.3) | 49 (39.5) | 0.296 | 96 (40.5) | 14 (26.9) | 0.068 |
| Tracheostomy, n (%) | 17 (4.3) | 12 (9.6) | 0.025 | 12 (5.0) | 1 (1.9) | 0.322 |
| Vasopressor support, n (%) | 332 (85.1) | 101 (81.4) | 0.328 | 195 (82.2) | 47 (90.3) | 0.151 |
| Renal replacement therapy, n (%) | 26 (6.6) | 10 (8.0) | 0.595 | 18 (7.5) | 2 (3.8) | 0.335 |
| ECMO, n (%) | 8 (2.0) | 1 (0.8) | 0.357 | 5 (2.1) | 0 (0) | 0.291 |
| ICU length of stay, median (IQR), days | 3 (1-6) | 2 (1-7) | 0.813 | 3 (1-6) | 2 (1-4) | 0.005 |
| Hospital length of stay, median (IQR), days ^e^ | 16 (8-28) | 18 (10-33) | 0.060 | 16 (8-29) | 17 (11-29) | 0.512 |
| Discharged to home from the hospital, n (%) ^f^ | 293 (75.7) | 87 (70.7) | 0.270 | 169 (71.9) | 41 (78.8) | 0.214 |

*A total of 11 patients had missing EQ-5D-5L data only at admission, 116 had it missing only at 6 months, and 3 had missing data at both time points, respectively.

**A total of 1 patient had missing Barthel index data only at admission, 52 had it missing only at 6 months, and 1 had missing data at both time points, respectively.

^a^ Of 514 participants, 2 were missing.

^b^ Of 514 participants, 130 were missing.

^c^ Of 514 participants, 116 were missing.

^d^ Of 514 participants, 2 were missing.

^e^ Of 514 participants, 2 were missing.

^f^ Of 514 participants, 4 were missing.

CFS, Clinical Frailty Scale; ICU, intensive care unit; IQR, interquartile range; APACHE2, acute physiology and chronic health evaluation 2; ECMO, extracorporeal membrane oxygenation.
